# Channel-cut monochromator withstanding incident powers above 400 W on undulator beamlines

**DOI:** 10.1107/S1600577525009695

**Published:** 2026-01-01

**Authors:** Hiroshi Yamazaki, Yasuhiro Shimizu, Koji Tsubota, Kazuhiko Tahara, Satsuki Shimizu, Takahisa Koyama, Hirokatsu Yumoto, Taito Osaka, Ichiro Inoue, Makina Yabashi, Haruhiko Ohashi

**Affiliations:** a Japan Synchrotron Radiation Research Institute 1-1-1 Kouto Sayo Hyogo679-5198 Japan; b RIKEN SPring-8 Center, 1-1-1 Kouto, Sayo, Hyogo679-5148, Japan; Advanced Photon Source, USA

**Keywords:** channel-cut monochromators, double channel-cut, cryogenic cooling, thermal deformation, beam stability, fixed-exit geometry, fourth-generation synchrotron radiation

## Abstract

A liquid-nitrogen-cooled silicon channel-cut monochromator was developed and experimentally evaluated under high-thermal-load conditions. Under the maximum load of 417 W, the first reflecting surface exhibited a concave deformation, resulting in only an 11% reduction in vertical beam size at 16 m downstream. The deformation radius was estimated at 510 m. Despite the deformation, no significant changes were observed in the angular profile or intensity of the monochromatic beam. Interference fringes caused by edge diffraction at an upstream slit confirmed excellent preservation of spatial coherence. For the stability test of the monochromator, intensity fluctuation of the monochromatic beam was monitored and linearly fitted with upstream beam-position monitor signals, which were synchronously acquired. A high correlation (*R*^2^ = 0.95) confirmed that the inherent stability of the channel-cut design remained under cryogenic cooling. Additionally, a double channel-cut monochromator configuration for fixed-exit beam operation was tested and produced the expected output beam intensity. These results confirm the feasibility of using channel-cut monochromators as high-stability high-heat-load-tolerant optical elements for next-generation synchrotron beamlines.

## Abstract 2

A liquid-nitrogen-cooled silicon channel-cut monochromator was developed and tested under synchrotron radiation with incident powers up to 417 W. The minimal deformation and the maintained high stability indicate its suitability as a first optical element for fourth-generation synchrotron sources, such as SPring-8-II.

## 1. Introduction

Silicon double-crystal monochromators (DCMs) and channel-cut monochromators (Bonse & Hart, 1965 ▸) are widely used as X-ray optical elements in synchrotron radiation beamlines. DCMs are relatively preferred as the first optical component due to the relative ease of fabricating and polishing their reflecting surfaces using well established mechanochemical techniques. In high-brightness synchrotron sources, however, thermal management becomes critical. Inadequate cooling can lead to deformation of the crystal surfaces (as demonstrated by Rutishauser *et al.*, 2013 ▸; Zhang *et al.*, 2013 ▸; Chumakov *et al.*, 2014 ▸; *etc*.), degrading beam quality. Cryogenic cooling using liquid nitrogen (LN2) is commonly employed, owing to its high thermal conductivity and low thermal expansion at cryogenic temperatures (Bilderback, 1986 ▸; Bilderback *et al.*, 2000 ▸). Despite their widespread use, DCMs are susceptible to relative angular misalignments between the two crystals, especially under cryogenic cooling. Vibrations induced by LN2 flow can independently disturb each crystal, impairing beam stability. Efforts are underway to suppress such vibrations to less than 50 nrad (Yamazaki *et al.*, 2013 ▸, 2019 ▸; Bai *et al.*, 2023 ▸).

Channel-cut monochromators, in contrast, provide excellent intrinsic angular stability because both reflecting surfaces are carved from a single silicon crystal, ensuring sub-atomic-level parallelism between the diffraction planes. Historically, their adoption was limited by difficulties in polishing internal surfaces and by restrictions on usable photon energies due to material size. However, recent advances in crystal growth and ultra-precision polishing have largely mitigated these limitations. Today, monolithic silicon crystals with diameters up to 150 mm are commercially available, and surface quality has improved significantly by plasma etching (Hirano *et al.*, 2019 ▸; Matsumura *et al.*, 2024 ▸).

One of the main remaining challenges is thermal management. The first reflecting surface absorbs the majority of incident power, leading to local thermal deformation. Channel-cut crystals, like the first crystal in DCMs, require efficient cooling. However, achieving strong thermal contact via tight clamping can introduce mechanical strain, potentially causing twisting between the two reflecting surfaces. Widely used weak links grooved between the two reflecting surfaces can reduce global angular mismatches by mechanical anti-twisting but have limited effect on beam quality when local deformation is present. Furthermore, channel-cut monochromators exhibit displacement of the exit beam during energy scans due to their fixed geometry. A promising solution is the use of a double channel-cut monochromator (DCCM) configuration, in which a second mirror-symmetric channel-cut crystal is placed downstream to provide a fixed exit. Although this design is intrinsically dispersive, the resulting intensity loss is mitigated in third- and fourth-generation synchrotron sources due to their low angular divergence.

This study aims to evaluate whether a liquid-nitrogen-cooled silicon channel-cut monochromator can withstand the high thermal loads typical of fourth-generation synchrotron sources while maintaining excellent beam quality and mechanical stability. In addition, we examine the performance of a DCCM configuration for fixed-exit operation and compare experimental results with theoretical predictions based on synchrotron radiation simulations and the DuMond analysis (DuMond, 1937 ▸).

## 2. Beamline equipment

A pair of silicon channel-cut monochromators were installed at beamline BL05XU (Yumoto *et al.*, 2020 ▸) at SPring-8. The light source was an in-vacuum planar undulator with 93 pairs of 32 mm-period magnets.

**Figure 1:**
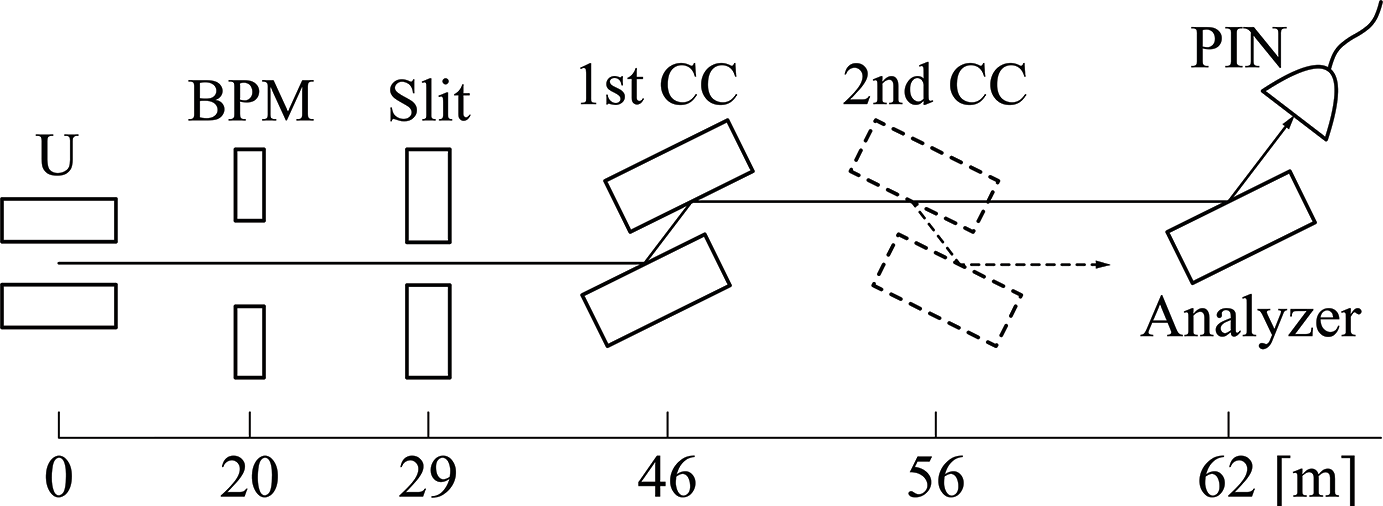
Beamline layout. The numbers indicate distances from the undulator (U). BPM: beam-position monitor; CC: channel cut. Monochromatic beams were analyzed at the experimental station located 62 m downstream, where a beam image monitor, a θ–2θ goniometer mounting an analyzer crystal and a PIN photodiode (illustrated in this figure), and a PIN photodiode were alternately installed for different measurements.

By adjusting the undulator gap, the fundamental photon energy was tunable between 4.5 keV (2.8 Å) and 15 keV (0.83 Å). Fig. 1 ▸ illustrates the beamline layout. The incident beam was monitored by a beam-position monitor (BPM) (Aoyagi *et al.*, 2020 ▸) located 20 m downstream from the source, using four off-axis tungsten electrodes to detect beam motion. A slit positioned at 29 m defined the beam size, with dimensions of up to 0.56 mm (vertical) and 1.86 mm (horizontal) in this study.

The first channel-cut monochromator was installed at 46 m in an upward-bounce geometry, enclosed within a vacuum chamber. It employed the Si 111 reflection with a Bragg angle of 26.3° at a photon energy of 4.46 keV (2.78 Å), corresponding to an undulator gap of 8.30 mm. Under this condition, the incident power on the crystal was calculated at 417 W using a synchrotron radiation calculator, *SPECTRA* version 12 (Tanaka & Kitamura, 2001 ▸; Tanaka, 2021 ▸). The peak power density reached 160 W mm^−2^ in the beam-normal direction. The crystal was mounted on a high-precision goniometer with an angular resolution of 0.1 arcsec (∼500 nrad). LN2 was circulated through vacuum-insulated transfer lines from an LN2 circulator (Suzuki Shokan Co. Ltd prototype) to cool the crystal. A tungsten beam stopper was placed immediately downstream to block directly transmitted X-rays. Further details of the crystal geometry and cooling assembly are provided in Section 3.

In the DCCM configuration, a second mirror-symmetric channel-cut crystal was installed at 56 m in a downward-bounce geometry. As the thermal load was significantly reduced after the first monochromator, the second crystal was operated at room temperature without any cooling.

The monochromated beam exited to the experimental station at 62 m, passing through beryllium and silicon nitride windows. Beam quality was characterized using three methods: (1) spatial profiling with a beam-image monitor (Kameshima *et al.*, 2019 ▸), (2) angular profiling via θ–2θ goniometry (Kohzu Precision Co. Ltd RA20-21-1V) and (3) intensity measurements with a silicon PIN photodiode (Hamamatsu Photonics KK S3590-09 packaged by Ohyo Koken Kogyo Co. Ltd). Each piece of equipment was alternately installed at the same position. For beam-stability assessment, BPM currents and photodiode signals were synchronously recorded at 1 kHz with 1 ms integration.

## 3. Crystal design and cooling assembly

**Figure 2:**
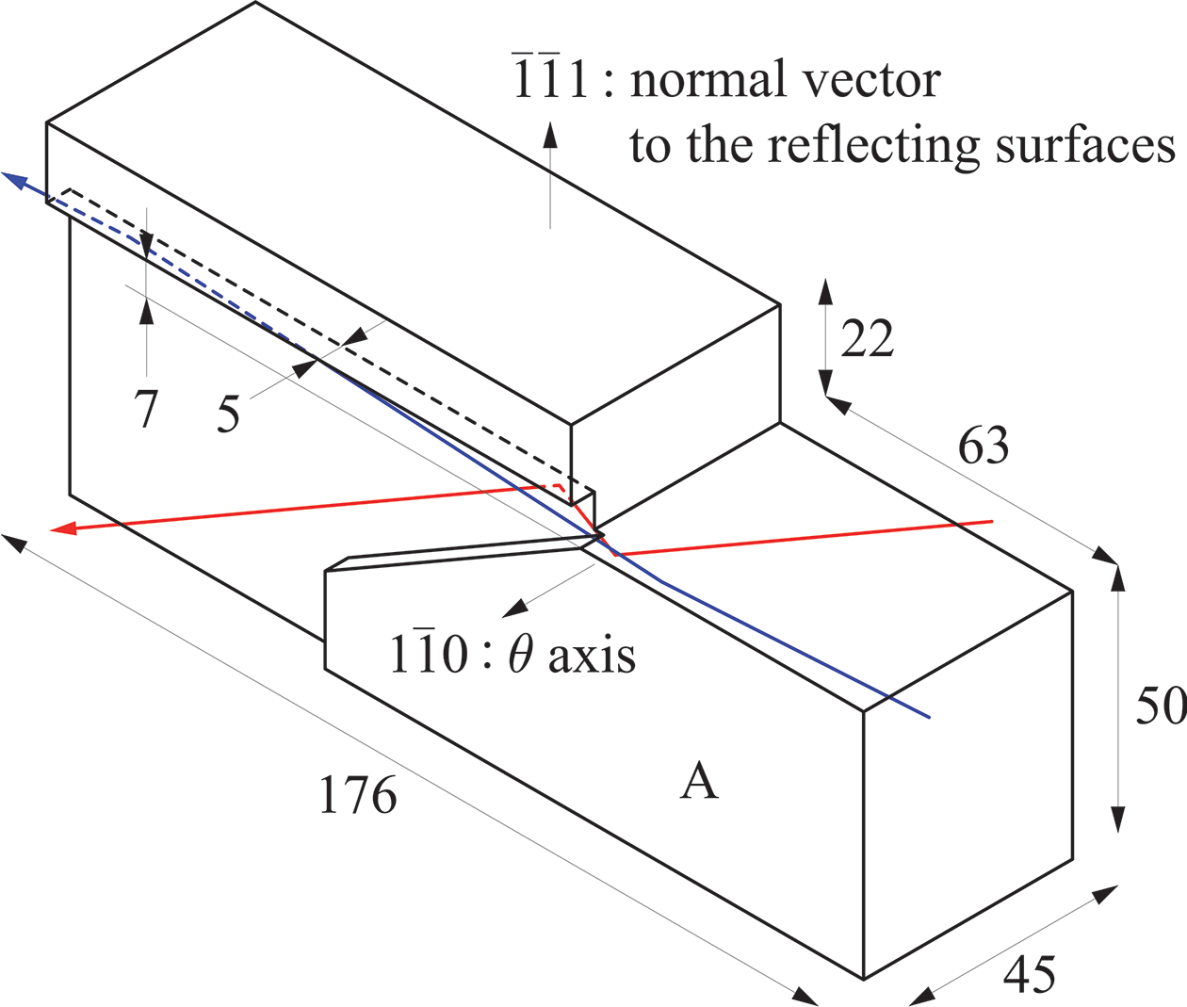
Design of the first channel-cut crystal. All dimensions are in millimetres. Bragg angles were varied by θ rotation of the crystal about the 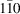 axis. Red and blue lines represent beam paths relative to the crystal orientation at photon energies of 4.46 and 24.8 keV, respectively.

The first channel-cut crystal (see Fig. 2 ▸) was fabricated from a 150 mm-diameter high-purity silicon ingot grown by the floating-zone method. Assuming a [111] growth direction, the 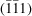 crystallographic plane was selected for the reflecting surfaces. Bragg angles were varied by rotating the crystal about the 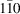 axis. To maintain structural rigidity, the channel was designed with a shallow depth of 5 mm and a fixed gap of 7 mm between the reflecting surfaces. The selected geometry enabled operation across a wide photon-energy range from 4.0 to 32 keV. Subsurface damage was removed by chemical etching. At the time of the experiment, plasma etching to smooth the reflecting surfaces had not yet been completed. Remaining scratches and voids may locally disturb beam images but bring negligible effects for the performance tests carried out in Section 4.

**Figure 3:**
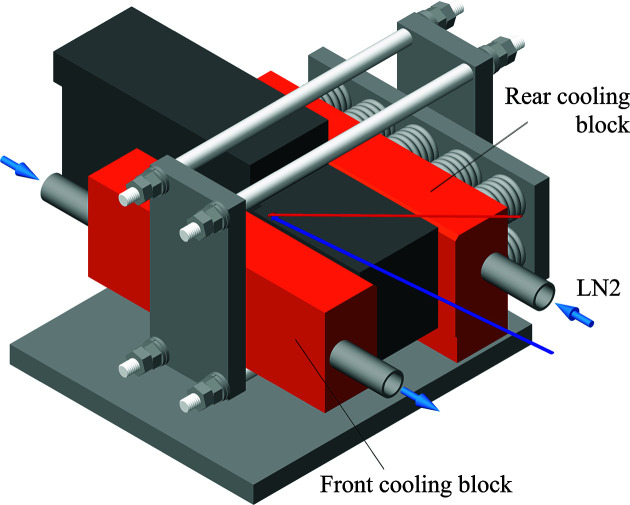
Cooling assembly of the first channel-cut crystal.

The two reflecting surfaces were fabricated specifically to accommodate the difference in thermal loads. The first surface, which received the majority of the incident radiation, featured a thick support body 50 mm in height (labeled A in Fig. 2 ▸) to ensure heat dissipation and mechanical stability. This body was tightly clamped between two copper cooling blocks, each containing LN2 channels 10 mm in diameter (see Fig. 3 ▸). To enhance thermal contact, the contact faces of the silicon were additionally flattened by mechanochemical polishing, and 0.1 mm-thick graphite sheets were inserted at each interface. Heat-load tests at SPring-8 confirmed that graphite sheets have superior thermal contact compared with conventional indium foils. LN2 flowed through the rear cooling block from the upstream to the downstream side of the beam, made a U-turn at the downstream end, and then flowed through the front cooling block from downstream to upstream.

As the front block was positioned adjacent to the irradiated region, it absorbed the majority of the heat, thereby allowing the rear side of the crystal to remain thermally stable across the operating range. In contrast, the second reflecting surface, subjected to minimal thermal loading, was left uncooled to avoid mechanical twisting due to asymmetric clamping forces. Assembly was performed at room temperature and mechanical compliance under cooling was ensured using coil springs. The LN2 flow rate was 7.7 L min^−1^, with an inlet temperature of 78.3 K.

**Figure 4:**
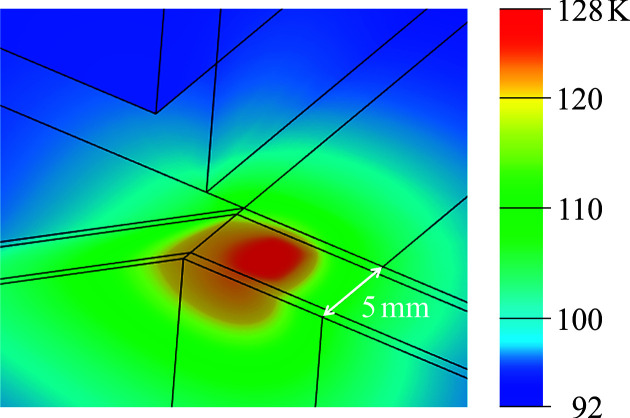
Simulated temperature distribution on the first reflecting surface under an incident power of 417 W.

Temperature distribution (see Fig. 4 ▸) was estimated using finite-element analysis performed with *ANSYS Mechanical 2024 R1* (https://www.ansys.com/en-gb/products/structures/ansys-mechanical), incorporating volumetric heat generation profiles calculated via *SPECTRA*. Of the 417 W incident power, ∼217 W was absorbed by the crystal. The convective heat transfer coefficient between the LN2 and copper blocks was estimated at 3900 W m^−2^ K^−1^ using the Dittus–Boelter equation (Winterton, 1998 ▸). The interfacial thermal conductance between the copper, graphite and silicon layers was set to 3000 W m^−2^ K^−1^, a conservative value within the range of 3000–8000 W m^−2^ K^−1^ estimated from the heat-load tests. The peak crystal temperature reached 128 K, which is near the zero thermal expansion point of silicon. Based on the simulated temperature profile, the thermal deformation resulted in a concave bump on the reflecting surface with an estimated radius of curvature of 490 m.

## 4. Experimental results

### 4.1. Thermal management

**Figure 5:**
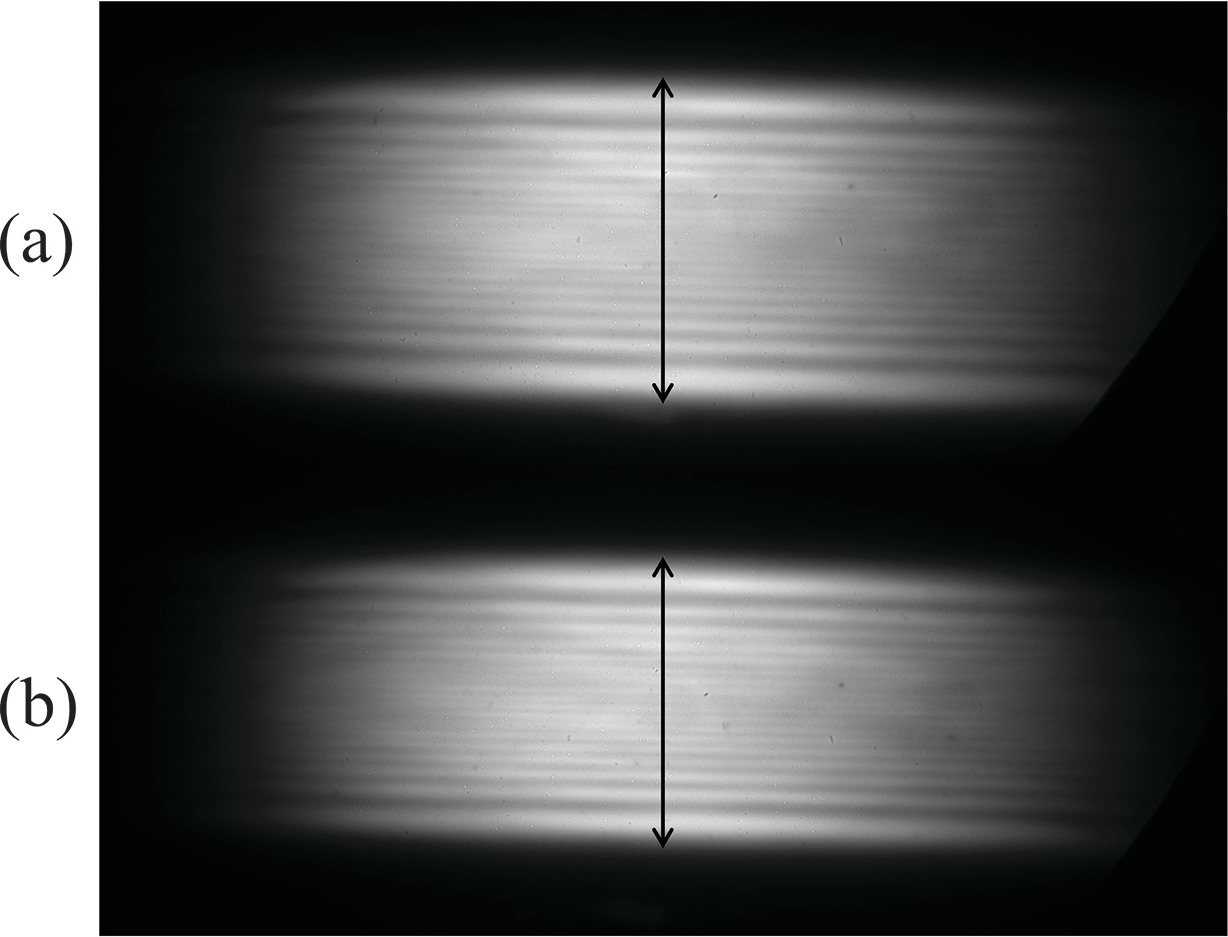
Beam images from the first channel-cut monochromator at ring currents of (*a*) 1 mA and (*b*) 100 mA. Arrows show the vertical beam sizes: (*a*) 1.13 mm and (*b*) 1.01 mm.

Monochromatic beams with a photon energy of 4.46 keV were recorded from the first channel-cut monochromator while varying the storage-ring current from 1 to 100 mA, corresponding to a maximum incident power of 417 W. As the current increased, the vertical beam size decreased from 1.13 to 1.01 mm, representing an 11% reduction (see Fig. 5 ▸), while the beam intensity linearly increased. This shrinkage is attributed to a thermally induced concave deformation on the first reflecting surface, which grew with increasing heat load.

To estimate the radius of curvature, a geometric ray model was employed under the assumption that the surface was flat at 1 mA. Given the distances from the source to the monochromator (46 m) and from the monochromator to the beam-image monitor (16 m), the radius at 100 mA was estimated to be ∼510 m. This result closely matched the simulated value of 490 m, indicating good agreement between model and observation.

Beam images exhibited fine horizontal interference fringes due to edge diffraction from the slit. The sharp contrast in these fringes suggests that the channel-cut monochromator preserved the beam’s transverse coherence well.

**Figure 6:**
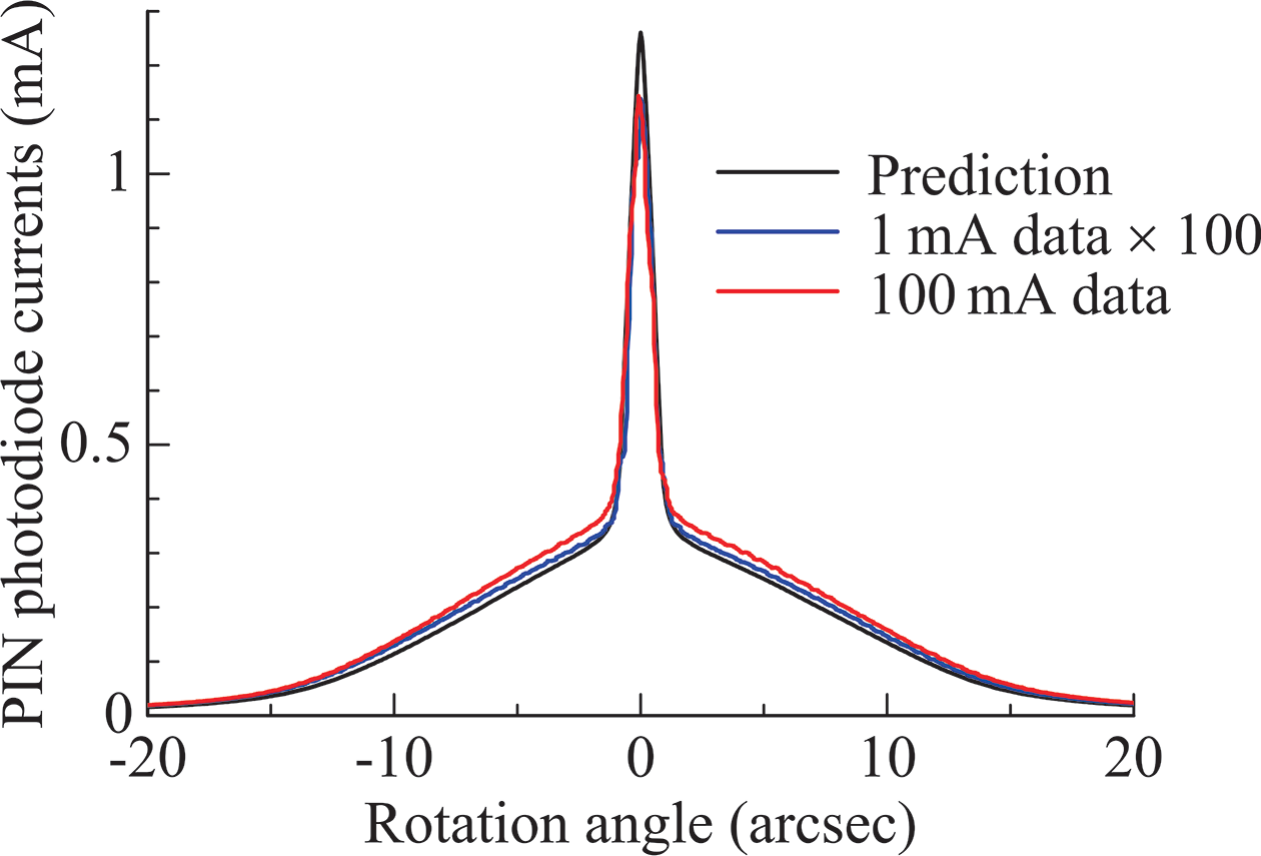
Measured angular profiles at the ring currents of 1 and 100 mA, and their prediction.

Angular profiles were obtained using a θ–2θ scan with an upward-bounce Si 111 analyzer crystal. The setup was in a non-dispersive geometry, free from chromatic broadening. The measured photodiode-current profiles, normalized by ring current, are shown in Fig. 6 ▸, together with the prediction based on the DuMond analysis assuming no thermal deformation. Each profile showed a broad base corresponding to the 111 reflection and a narrower central peak resulting from higher-order-harmonic contamination (333 reflection, photon energy 13.4 keV). The prediction included the effects from the angular-dependent spectrum of radiation and the finite incident slit aperture, both of which were computed using *SPECTRA*. By taking into consideration the absorption by the exit windows and air, the peak fluxes were predicted at 4.0 × 10^13^ and 2.9 × 10^12^ photons s^−1^ for the 111 and 333 reflections, respectively. Since sensitivity of the photodiode depended on photon energy, the fluxes were converted into the photodiode current to compare with the experimental profiles. No significant difference between the experimental profiles was observed within the resolution of this angular-profiling method, even for the higher-order reflection.

### 4.2. Mechanical stability

Although channel-cut crystals are inherently stable, their mechanical stability must still be experimentally verified under realistic operating conditions. Fluctuations in the intensity of a monochromatic beam can originate not only from instabilities in a channel-cut crystal but also from variations in the incident beam. For a proper stability assessment, the latter contribution has to be separated.

To address this, we used the upstream BPM, which was originally designed to track incident-beam motion, to determine what fraction of the downstream intensity fluctuations can be explained by the BPM currents. As the simplest regression model, a first-order linear expression was constructed using the BPM currents *x*_*i*_ (*i* = 1, 2, 3, 4) as explanatory variables,
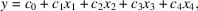
where *y* represents the photodiode current. The five coefficients *c*_0_ and *c*_*i*_ were determined via least-squares optimization using a large dataset to ensure statistical robustness and to prevent overfitting. The resulting coefficient of determination, *R*^2^, indicates the fraction of the intensity fluctuations that can be explained by the BPM currents, which represent variations in the incident beam. Since the BPM currents are unrelated to the properties of channel-cut crystal, the remaining variance, 1 − *R*^2^, is attributed to the instability in the channel-cut crystal and other unaccounted factors.

The evaluation was performed at a photon energy of 24.8 keV (wavelength 0.50 Å), where the angular acceptance (Darwin width) of the Si 111 reflection is 2.98 arcsec, much narrower than at 4.46 keV (13.6 arcsec). This condition increases sensitivity to angular deviations, thereby providing a stringent test of stability. The undulator was tuned to select the third harmonic and the slit aperture was reduced to 0.3 mm × 0.3 mm to minimize thermal deformation effects.

**Figure 7:**
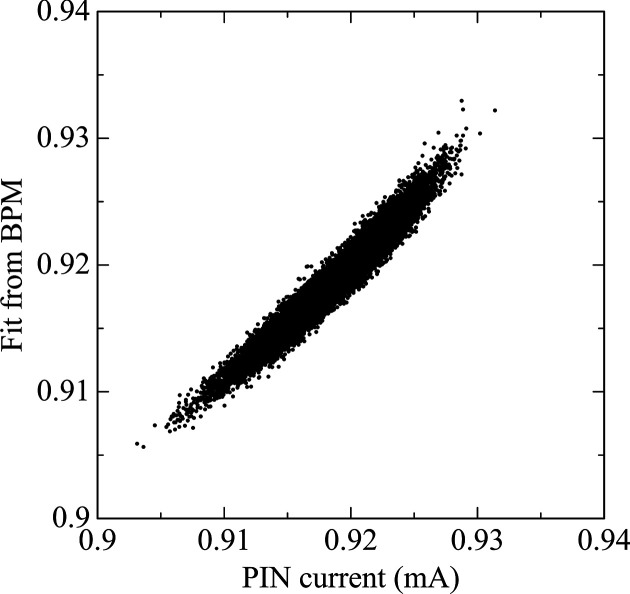
Correlation between measured intensity with the PIN photodiode and the fitted intensity from BPM signals.

**Figure 8:**
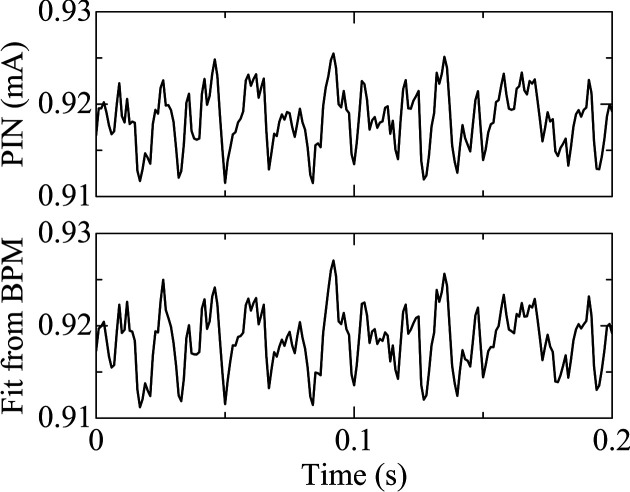
Comparison of measured and fitted beam intensities over a representative 0.2 s time window.

Beam intensity was measured over a 30 s period, at 1 kHz sampling rate, yielding a 30000-point dataset synchronized with corresponding BPM currents. The linear model showed a strong correlation with the measured photodiode signals (see Fig. 7 ▸), achieving *R*^2^ = 0.95. This result indicates that 95% of the intensity fluctuations can be explained by the variations in the incident beam, supporting the conclusion that the channel-cut monochromator remained highly stable under cryogenic conditions. A representative 0.2 s time window is shown in Fig. 8 ▸.

### 4.3. Performance in DCCM configuration

Rocking curves in the DCCM configuration were measured by rotating the second channel-cut crystal. Prior to this measurement, special attention was paid to tuning the undulator gap. Since undulator radiation exhibits angular-dependent spectra, gap values largely affect rocking-curve profiles. As a practical procedure, after adjusting the slit aperture and the DCCM angles, fine tuning of the undulator gap was carried out so that the intensity of the monochromatic beam could be maximized. In this measurement, the slit aperture was set to 0.56 mm (vertical) and 1.86 mm (horizontal).

**Figure 9:**
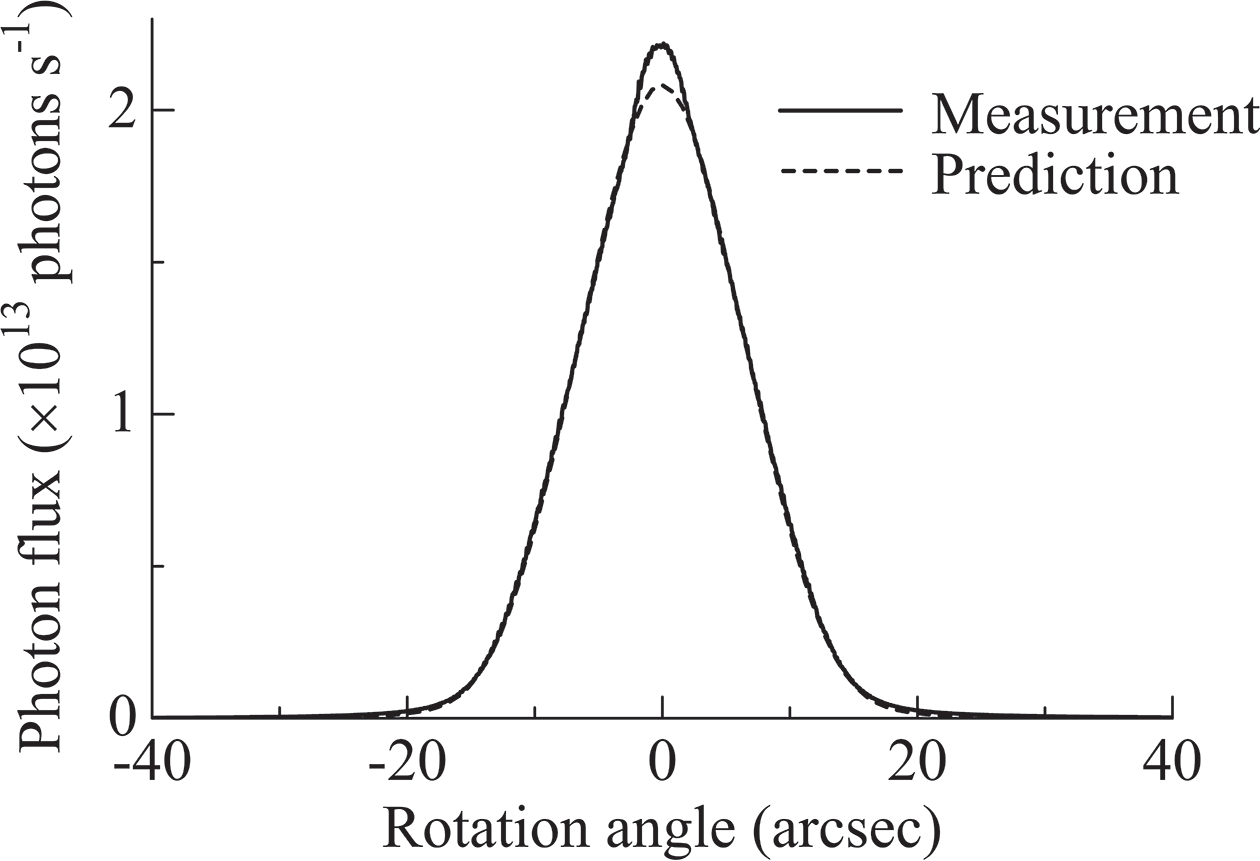
Measured and predicted rocking curves at 4.46 keV in the DCCM configuration.

At a photon energy of 4.46 keV, the measured rocking curve showed good agreement with the theoretical prediction based on DuMond analysis (see Fig. 9 ▸). The prediction included absorption effect by the exit windows. Unlike the angular profiles shown in Fig. 6 ▸, the measured rocking curve exhibited no visible peaks arising from higher-order-harmonic contamination. This absence was attributed to the significant suppression of higher-order-harmonic components in the dispersive (mirror-symmetry) geometry between the two channel-cut crystals. In the prediction, the peak fluxes were 2.1 × 10^13^ and 3.3 × 10^11^ photons s^−1^ for 111 and 333 reflections, respectively.

**Figure 10:**
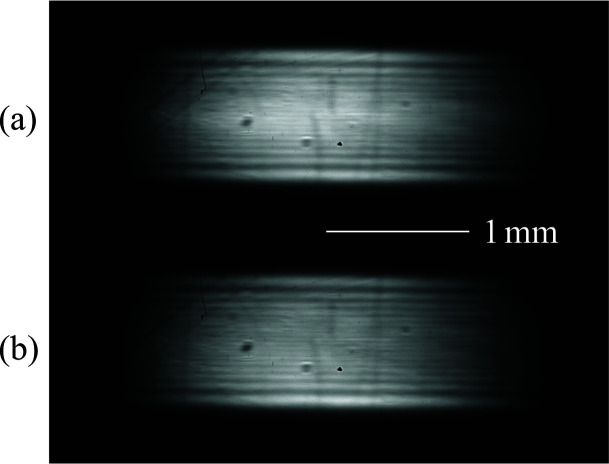
Beam images at 4.46 keV in the DCCM configuration: (*a*) at the peak angle and (*b*) after rotating the second monochromator by 0.1 arcsec.

The beam image at the peak angle closely resembled that obtained from the first channel-cut monochromator, except for a haze observed around the center of the beam height (see Fig. 10 ▸). This haze disappeared upon rotating the second crystal by 0.1 arcsec, confirming the presence of residual higher-order-harmonic contamination.

**Figure 11:**
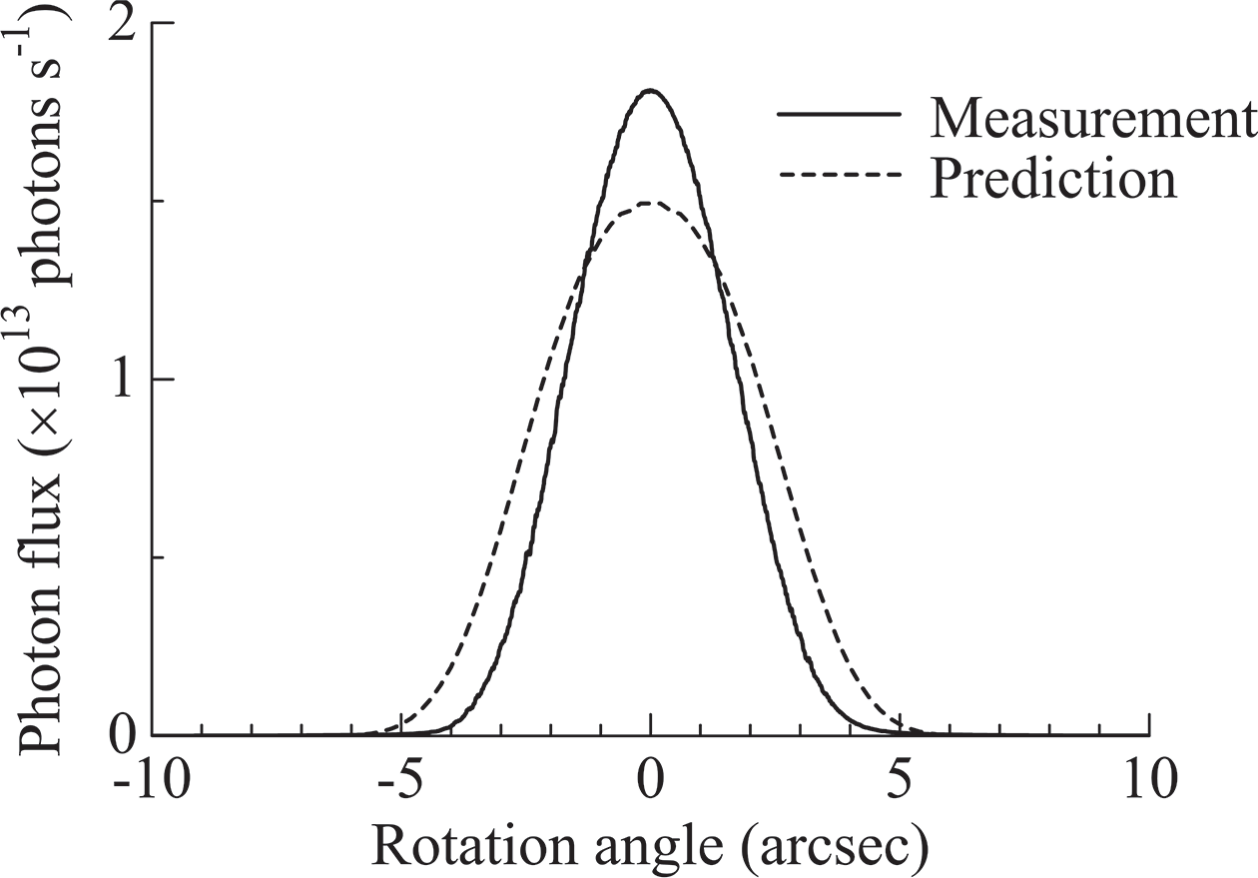
Measured and predicted rocking curves at 24.8 keV in the DCCM configuration.

A similar measurement was conducted at a photon energy of 24.8 keV (see Figs. 11 ▸ and 12 ▸). In this case, the measured rocking curve appeared slightly higher and narrower than predicted. Since monochromator imperfections typically result in broader and weaker peaks, this discrepancy is probably not due to crystal defects. It is more plausibly attributed to uncertainties in the model parameters used in the simulation. The observed oval-shaped beam profile at this energy is consistent with the narrow angular acceptance of the monochromators at high photon energies.

## 5. Discussion

A channel-cut monochromator has been developed to withstand incident heat loads up to 417 W while maintaining excellent intrinsic stability. This achievement is primarily attributed to two key design features: a specific channel-cut structure and a cooling assembly that concentrate on the first reflecting plane while avoiding mechanical twisting. The performance of the monochromator was validated by consistency between experimental observations and analytical simulations. Scratches and voids on the reflecting surfaces, which caused local inhomogeneities in the beam images, can be eliminated by plasma etching.

The manageable heat load directly affects the adaptability of the monochromator to advanced light sources. The forthcoming SPring-8-II (Tanaka *et al.*, 2024 ▸), scheduled to begin operation in 2029, features both horizontal and vertical low emittance. SPring-8-II will produce radiation more tightly focused at the fundamental undulator energy, enabling the use of narrower slit apertures. Simulations using in-vacuum undulators for SPring-8-II (IVU-IIs) (Imamura *et al.*, 2024 ▸) predict that the incident heat loads will be reduced to below 330 W at a photon energy of 4.0 keV when using slit apertures that transmit 86% (D4σ or second-moment width) of the fundamental radiation (Ohashi *et al.*, 2025 ▸). Therefore, the predicted heat loads are within the capability of the present design.

The higher-order-harmonic contamination in the monochromated beams confirmed the excellence of the monochromator’s performance. On the other hand, for experiments that are sensitive to the contamination of higher-order harmonics, the use of higher-order suppression mirrors is required because channel-cut crystals have no freedom for detuning.

The observed interference fringes, caused by edge diffraction at the slit, indicate that the channel-cut monochromators effectively preserve the spatial coherence derived from the small source size. The suppression of beam inhomogeneity caused by high spatial coherence is a challenging issue for high-precision applications.

The radius of the thermal bump was experimentally estimated using a geometric ray model, as described in Section 4.1. While this estimation is considered a reasonable first approximation, its validity should be further confirmed using dynamical diffraction theory, given the volumetric nature of X-ray diffraction in crystals, unlike the surface-based reflection in mirrors.

## 6. Conclusions

A liquid-nitrogen-cooled silicon channel-cut monochromator was developed to withstand incident thermal loads up to 417 W. This performance was achieved through two key design features: a specific channel-cut geometry and a cooling assembly optimized to cool the first reflecting surface while minimizing mechanical twisting. Under maximum thermal load, the thermally induced concave deformation was well suppressed, with an experimentally measured deformation radius of ∼510 m, consistent with the simulated value of 490 m. This deformation introduced a mild focusing effect, reducing the vertical beam size by only 11% at 16 m downstream. Importantly, no significant degradation in angular profile, beam intensity or spatial coherence was observed. The inherent intensity stability of the channel-cut design was preserved under cryogenic conditions, with the monochromatic beam intensity exhibiting a strong correlation (*R*^2^ = 0.95) with the incident beam, indicating that 95% of the observed fluctuations could be attributed to upstream variations.

A DCCM configuration for fixed-exit beam operation was also evaluated. The measured beam intensity was consistent with predictions from synchrotron radiation simulations and DuMond analysis, validating the performance of the design.

The demonstrated thermal-load tolerance of 417 W exceeds the anticipated heat loads (∼330 W) expected for the first optical elements in the forthcoming SPring-8-II standard undulator beamlines. This is enabled in part by the low horizontal emittance of the SPring-8-II electron beam, which produces highly focused radiation and permits the use of narrow slit apertures.

In summary, these results support the feasibility of using channel-cut monochromators as optical components with high stability and high heat-load tolerance for next-generation synchrotron beamlines. As an application example, a liquid-nitrogen-cooled DCCM has been installed and been in operation at BL40XU since April 2025, handling moderate incident powers (<30 W). Fixed-exit operation is achieved by inserting or retracting the DCCM to switch between beams with narrow (∼0.01%) and wide (∼2%) energy bandwidths.

## Figures and Tables

**Figure 12:**
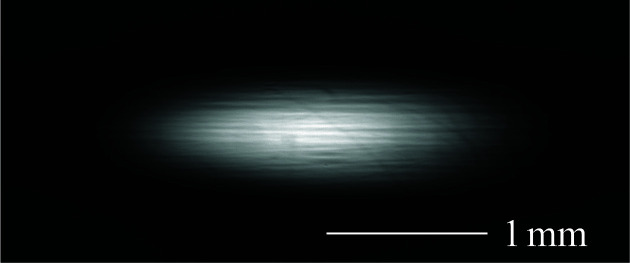
Beam image at 24.8 keV in the DCCM configuration, captured at the peak angle.

## Data Availability

The data supporting this study are available from the corresponding author upon reasonable request.
