# Channel-cut monochromator withstanding incident powers above 400 W on undulator beamlines. Corrigendum

**DOI:** 10.1107/S1600577526001116

**Published:** 2026-02-17

**Authors:** Hiroshi Yamazaki, Yasuhiro Shimizu, Koji Tsubota, Kazuhiko Tahara, Satsuki Shimizu, Takahisa Koyama, Hirokatsu Yumoto, Taito Osaka, Ichiro Inoue, Makina Yabashi, Haruhiko Ohashi

**Affiliations:** a Japan Synchrotron Radiation Research Institute 1-1-1 Kouto Sayo Hyogo679-5198 Japan; b RIKEN SPring-8 Center, 1-1-1 Kouto, Sayo, Hyogo679-5148, Japan; Advanced Photon Source, USA

**Keywords:** channel-cut monochromators, double channel-cut, cryogenic cooling, thermal deformation, beam stability, fixed-exit geometry, fourth-generation synchrotron radiation

## Abstract

Fig. 2 in the article by Yamazaki *et al.* [(2026). *J. Synchrotron Rad.***33**, 84–90] contained an inconsistency that has been corrected without affecting the results or conclusions.

## Abstract 2

Corrigendum to the article by Yamazaki *et al.* [(2026). *J. Synchrotron Rad.***33**, 84–90].

In the originally published version of Fig. 2 ▸ in Yamazaki *et al.* (2026 ▸), the beam paths were not consistent with the photon energies specified in the figure caption, which could lead to a misleading interpretation. Fig. 2 ▸ has been replaced with a revised version having consistent beam paths and auxiliary lines to clarify the geometrical relation used in the thermal estimation.

## Figures and Tables

**Figure 2:**
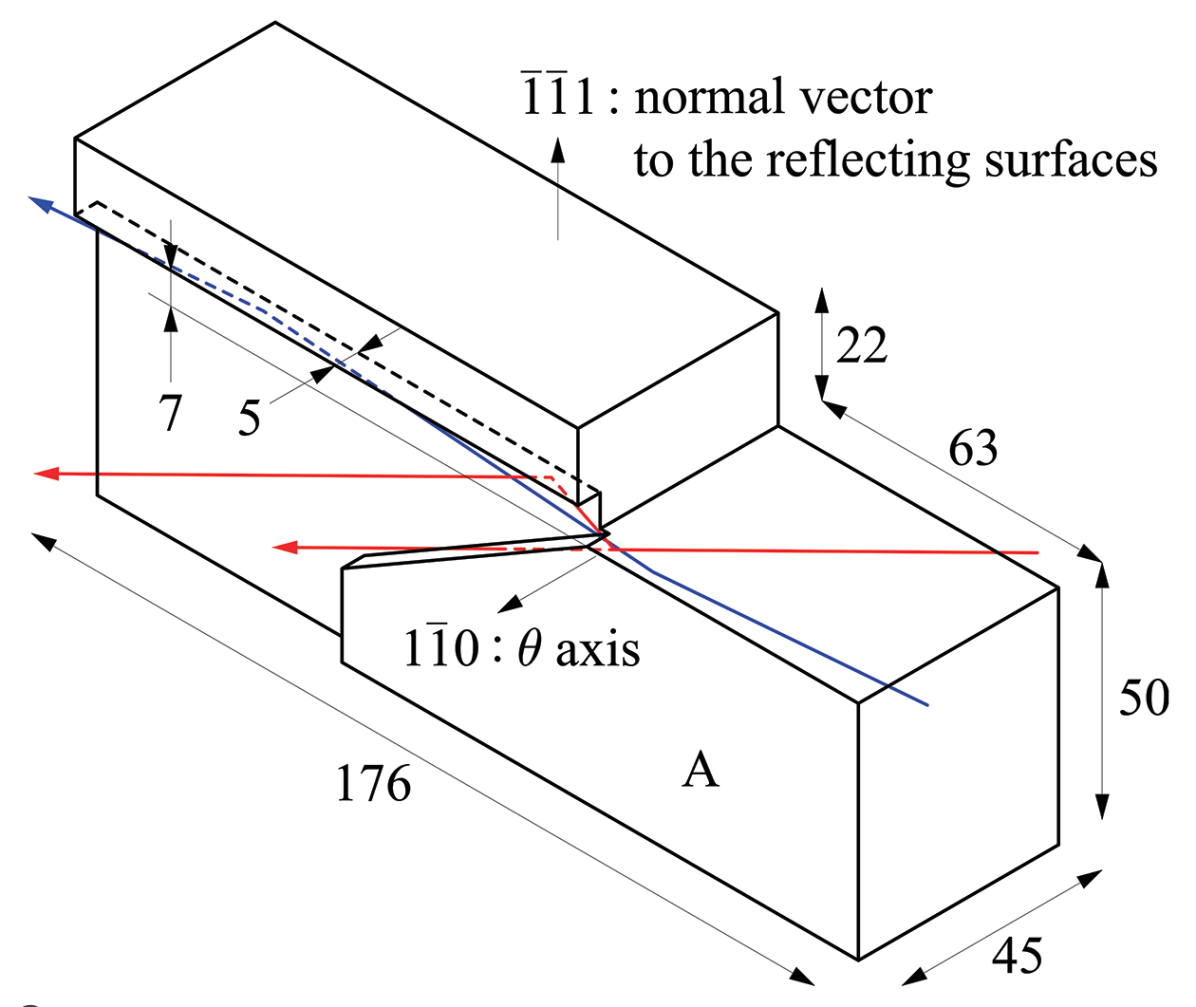
Design of the first channel-cut crystal. All dimensions are in millimetres. Bragg angles were varied by θ rotation of the crystal about the 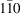 axis. Red and blue lines represent beam paths relative to the crystal orientation at photon energies of 4.46 keV and 24.8 keV, respectively. Higher-energy components in the incident beam escape from the side surface of the first reflecting body.
